# Early in-hospital fasting blood glucose and 90-day functional outcome in early-onset ischaemic stroke: a multicentre prospective cohort study

**DOI:** 10.3389/fneur.2026.1895654

**Published:** 2026-10-30

**Authors:** Yanfei Pan, Qing Peng, Luhua Wei, Wei Sun, Ran Liu, Haiqiang Jin, Weiping Sun, Haiying Xing, Yining Huang, on behalf of the CASTOR Investigators

**Affiliations:** Department of Neurology, Peking University First Hospital, Beijing, China

**Keywords:** early-onset ischaemic stroke, fasting blood glucose, functional outcome, risk stratification, young adults

## Abstract

**Background:**

The prognostic value of fasting blood glucose (FBG) in early-onset ischaemic stroke and its variation by prior diabetes status remain uncertain.

**Methods:**

We analysed 1,127 patients aged 18–49 years from the prospective multicentre CASTOR registry. For study eligibility, acute ischaemic stroke was defined as ischaemic stroke with admission within 7 days of symptom onset; thrombolysed patients were admitted within the thrombolytic time window. FBG was the first fasting venous glucose value from routine post-admission biochemistry; sampling time was unavailable. Unfavourable outcome was defined as a 90-day modified Rankin Scale score ≥3. Missing laboratory data underwent multiple imputation by chained equations. Multivariable logistic regression assessed associations, restricted cubic splines evaluated non-linearity, and interaction with prior diabetes was tested. Incremental predictive value was assessed by comparing models with and without FBG. Internal validation used repeated stratified 70/30 splitting, repeated five-fold cross-validation and bootstrap optimism correction.

**Results:**

Median age was 45.01 years (IQR 40.91–47.91); 902 patients (80.0%) were male. The median National Institutes of Health Stroke Scale score assessed within 24 h of admission was 2 (IQR 1–4). Unfavourable outcomes occurred in 283 patients (25.1%). Each 1 mmol/L higher FBG was associated with higher adjusted odds of an unfavourable outcome (OR 1.30, 95% CI 1.15–1.46). There was no evidence of non-linearity (pooled Wald *p* = 0.992) or interaction with prior diabetes (interaction OR 0.87, 95% CI 0.69–1.09; *p* = 0.220), although only 200 patients had prior diabetes (57 events), limiting power to detect interaction. The FBG association was consistent across sensitivity analyses and internal validation. Adding FBG increased the area under the receiver operating characteristic curve by a median 0.024 (median DeLong *p* = 0.025) across five imputed datasets, but the event component of continuous net reclassification improvement was negative (−0.035).

**Conclusion:**

Higher baseline FBG was independently associated with worse 90-day outcome, but its modest, inconsistent incremental predictive value does not establish utility for individual risk stratification. External validation with standardised sampling times is required.

## 1 Introduction

Stroke in young adults is increasingly recognized as a growing clinical and public health problem (1–3). Although stroke predominantly affects older populations, temporal trends in stroke incidence have been less favourable in younger than in older adults, with increases reported in younger populations while incidence at older ages has generally declined (4, 5). Young adults who suffer a stroke face disruptions to employment, social participation, long-term cardiovascular health, and psychosocial wellbeing that can persist for decades (6–9).

The risk factor profile of early-onset ischaemic stroke (EOS) overlaps only partially with that of stroke in later life (10–12). Traditional vascular risk factors, including hypertension, diabetes, dyslipidemia, smoking, obesity, and elevated BMI, are common and increasingly recognized in young stroke populations, but their relative contribution differs by age, sex, geography, and stroke mechanism (10, 11, 13). EOS also includes a broader spectrum of etiologies, such as patent foramen ovale, cervical artery dissection, autoimmune disease, prothrombotic disorders, and other uncommon causes (11, 14, 15).

Among metabolic disturbances, acute hyperglycemia has attracted particular attention. In unselected acute ischaemic stroke populations, elevated blood glucose has been repeatedly associated with greater neurological severity, larger infarct volumes, and worse functional recovery (16–20). More recent work has examined relative glycemic indices such as the stress hyperglycemia ratio, underscoring the prognostic relevance of acute glycemic perturbation superimposed on chronic metabolic status (21–25).

Whether these associations apply similarly to EOS remains incompletely established. The evidence base specific to younger stroke patients is limited and heterogeneous (14, 22, 26, 27). In the Korean SKY cohort, more severe initial symptoms, higher initial glucose, and systemic lupus erythematosus were associated with unfavourable outcome (27). In an endovascular-treatment cohort stratified by age, fasting glucose predicted poor outcome in older but not younger patients (26). These findings suggest that early glycemic markers may carry prognostic information in young stroke, but their effect size and interpretation may depend on age, diabetes status, stroke mechanism, and treatment context.

Importantly, randomized evidence has not established clinical benefit from intensive glucose lowering after acute ischaemic stroke. SHINE found no improvement in 90-day functional outcome with intensive glucose control (28, 29), whereas GIST-UK, stopped early because of slow recruitment, found no significant reduction in 90-day mortality or clear clinical benefit (30). Current AHA/ASA guidance treats inpatient glucose management as supportive rather than outcome-modifying (31). The principal clinical utility of early glycemic markers therefore lies in risk stratification, not treatment intensification.

In this study, we leveraged data from the prospective multicentre Chinese Acute Ischemic Stroke Treatment Outcome Registry (CASTOR) to examine the independent association between baseline fasting blood glucose and 90-day functional outcome in 1,127 adults aged 18–49 years with acute ischaemic stroke, consistent with the age range used in previous young-stroke cohorts (32, 33). We further explored whether pre-existing diabetes history modified this association.

## 2 Methods

### 2.1 Study design and population

This secondary analysis used data from the Chinese Acute Ischemic Stroke Treatment Outcome Registry (CASTOR), a prospective, multicentre, hospital-based registry that enrolled patients with acute ischaemic stroke from 80 hospitals across China between May 2015 and October 2017. The registry design and data-collection procedures have been reported previously (34, 35). We included adults aged 18–49 years with acute ischaemic stroke diagnosed according to the Chinese Guideline for Diagnosis and Treatment of Ischaemic Stroke, with thrombolysed patients admitted within the thrombolytic time window and non-thrombolysis patients admitted within 7 days of symptom onset. Patients were excluded if they had haemorrhagic transformation on baseline imaging, a life-limiting comorbidity with an expected survival of less than 3 months, or missing 90-day outcome data. The selection process is shown in Figure 1. The study was approved by the institutional review board of Peking University First Hospital (approval number: 2015[922]). Written informed consent was obtained from each patient or, when necessary, from a legally authorised representative.

**Figure 1:**
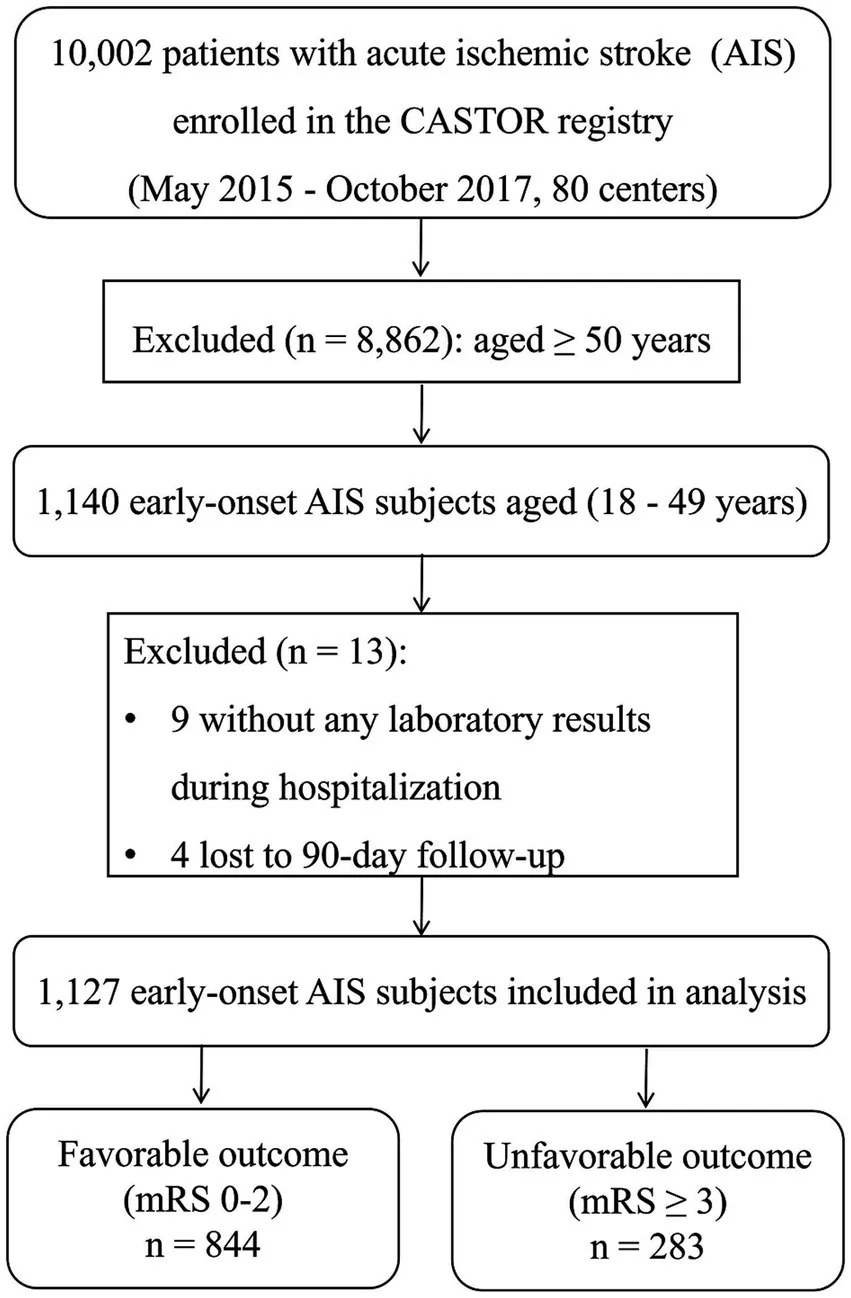
Flow diagram of participant selection from the CASTOR registry. Boxes report the number of patients excluded at each step and the corresponding reasons. AIS, acute ischaemic stroke; CASTOR, Chinese Acute Ischemic Stroke Treatment Outcome Registry; mRS, modified Rankin scale.

No separate *a priori* sample-size calculation was performed for this secondary analysis. All CASTOR participants aged 18–49 years who met the eligibility criteria and had available 90-day outcome data were included. This cohort analysis was reported in accordance with the Strengthening the Reporting of Observational Studies in Epidemiology (STROBE) statement (36).

### 2.2 Clinical variables and laboratory assessment

Baseline data encompassed demographics (age, sex), vascular risk factors (hypertension, diabetes mellitus, dyslipidemia, coronary heart disease, atrial fibrillation, prior stroke, carotid plaque), pre-stroke medications (aspirin, clopidogrel, statins, antihypertensives), and acute treatment variables. Neurological severity was assessed within 24 h of admission using the National Institutes of Health Stroke Scale (NIHSS) and categorized as mild (0–5), moderate (6–10), or severe (>10). Level of consciousness was assessed with the Glasgow Coma Scale (GCS) and categorized as 13–15, 9–12, or ≤8.

Laboratory testing was collected as part of the baseline in-hospital assessment in the CASTOR registry. The primary exposure in the present analysis was FBG (mmol/L), as recorded in the CASTOR electronic case report form. Additional laboratory variables included triglycerides (TG, mmol/L), total cholesterol (TC, mmol/L), high-density lipoprotein cholesterol (HDL-C, mmol/L), and low-density lipoprotein cholesterol (LDL-C, mmol/L). The TyG index was computed as ln[(TGmmol/L*88.57)*(FBGmmol/L*18.018)/2] to reflect insulin resistance.

FBG was measured in the clinical laboratory of each participating centre as part of the routine baseline biochemistry panel. In accordance with routine practice at the participating stroke units, this panel was obtained by venepuncture in the early morning after an overnight fast, typically on the first morning after admission; fasting status was therefore determined by routine clinical care and was not verified by a study-specific protocol. The registry recorded the FBG value but not the clock time of sampling, the interval from symptom onset to sampling, or the interval from admission to sampling, and these intervals therefore cannot be reported. Because patients who did not receive thrombolysis could be enrolled up to 7 days after symptom onset, the onset-to-sampling interval varied between patients, whereas the admission-to-sampling interval would be expected to be shorter and less variable, although this could not be verified. Whether the sample preceded the administration of intravenous fluids or of in-hospital insulin was likewise not captured in the electronic case report form. The only glucose-lowering treatment recorded in CASTOR was in-hospital therapy; because its timing relative to blood sampling was not recorded, it may have preceded the fasting sample in some patients, and its influence was examined in a sensitivity analysis. The exposure should therefore be interpreted as the first routinely obtained early in-hospital fasting glucose value rather than as a protocol-standardised measurement at a fixed interval after stroke onset.

### 2.3 Outcome assessment

The primary outcome was an unfavourable 90-day functional outcome, defined as a modified Rankin Scale (mRS) score of 3 or higher. The 90-day mRS was assessed by trained research staff at an in-person follow-up visit or by a standardised telephone interview.

### 2.4 Statistical analysis

All analyses were performed in R version 4.3.3. Continuous variables were summarised as median (Q1, Q3), and categorical variables as *n* (%). Continuous variables were winsorised at the 1st and 99th percentiles to reduce the influence of extreme values. For the descriptive summaries and between-group comparisons of age and admission NIHSS, original observed values were used without winsorisation or imputation. Baseline characteristics were compared between outcome groups using two-sided Wilcoxon rank-sum tests for continuous variables and the *χ*^2^ or Fisher exact test for categorical variables. Absolute standardized mean differences (SMDs) were used to quantify differences between the 90-day outcome groups, with values ≥0.10 considered potentially meaningful. For continuous variables, SMDs were calculated as the absolute between-group mean difference divided by the square root of the average of the two sample variances. For multilevel categorical variables, the maximum absolute level-specific SMD was reported. These SMDs were interpreted descriptively because the groups were defined by the subsequent outcome rather than by treatment assignment. Propensity-score matching or weighting was not used because the study estimated the prognostic association of a continuous baseline marker rather than the causal effect of an assigned treatment.

Among the analysis variables, missing data were confined to laboratory variables. FBG had the highest missingness (28.5%), followed by TG (22.6%). Body mass index was available but missing in 72% of patients and was therefore excluded. Smoking status and alcohol use were not collected in CASTOR. Missing laboratory data were handled using multiple imputation by chained equations. Five imputed datasets were generated for the primary analysis, and a 30-dataset re-imputation was performed as a sensitivity analysis. Because FBG was the exposure and the variable with the highest missingness, demographic, clinical, severity and treatment characteristics were additionally compared between patients with and without an observed FBG value using absolute standardized mean differences. All analysis variables, including the outcome, were included in the imputation model, so that the missing-at-random assumption is conditional on this information (37). The mechanism of missingness was characterised further by fitting a logistic model of FBG availability on all completely observed baseline variables and the 90-day outcome.

Potential non-linearity in the association between FBG and outcome was assessed using a four-knot restricted cubic spline, with knots at the 5th, 35th, 65th and 95th percentiles of FBG (FBG values of 4.09, 5.04, 5.99 and 10.37 mmol/L). Knot positions and the reference value were determined in the first completed dataset and the same positions were applied to every imputation, so that the spline coefficients and the pointwise contrasts were directly poolable. Non-linearity was evaluated using likelihood-ratio tests within each imputed dataset and a pooled Wald test of the nonlinear spline terms.

Candidate covariates for the primary model were initially screened using pooled univariable logistic regression analyses across the imputed datasets and were subsequently reviewed for clinical relevance, temporal ordering and redundancy, consistent with purposeful variable-selection and clinically informed covariate-selection principles (38, 39). NIHSS and GCS categories were retained as established indicators of neurological severity and level of consciousness, respectively (40, 41). Prior diabetes history and triglycerides were included to represent chronic glycaemic and metabolic status (20, 42). ICU admission was retained as an indicator of intensive care requirement, whereas pre-stroke aspirin use (43, 44), post-stroke HUK treatment (34, 45) and acute thrombolysis (31) were included to account for observed differences in prior treatment, acute management and potential treatment-selection effects. The final primary adjustment set included NIHSS category, GCS category, prior diabetes history, triglycerides, ICU admission, pre-stroke aspirin use, post-stroke HUK treatment and acute thrombolysis. The adjustment set was chosen to estimate the adjusted association of FBG with 90-day outcome. Coefficients for the remaining covariates are reported for completeness only and are not interpretable as causal effects of those variables, because each would require its own adjustment set; this applies in particular to prior diabetes history, for which FBG is an intermediate variable (46).

Post-stroke glucose-lowering therapy was excluded from the primary model because it is largely a clinical response to hyperglycaemia and could therefore represent an intermediate or treatment-selection variable; in addition, its timing relative to FBG sampling was not recorded (Section 2.2). Adjustment for a post-exposure intermediate may introduce overadjustment bias (47). Its inclusion was therefore evaluated separately in a sensitivity analysis.

Three pooled logistic regression analyses were performed: a main adjusted model in the full cohort, an interaction model including an FBG × prior diabetes term and a sensitivity analysis restricted to patients without prior diabetes. Additional sensitivity analyses used a DAG-minimum model, an extended model including age and sex, a model additionally including post-stroke glucose-lowering therapy, re-imputation with 30 datasets and complete-case analysis. Results are presented as odds ratios (ORs) with 95% confidence intervals (CIs).

Model discrimination was assessed using the area under the receiver operating characteristic curve (AUC). The full model included FBG, NIHSS category, GCS category, prior diabetes history, triglycerides, ICU admission, pre-stroke aspirin use, post-stroke HUK treatment and acute thrombolysis; the reduced model contained the same covariates except FBG. Because discrimination and reclassification metrics after multiple imputation do not have a universally accepted Rubin-style pooling approach, AUC, ΔAUC, DeLong *p* values, continuous net reclassification improvement (cNRI), and integrated discrimination improvement (IDI) were calculated separately within each imputed dataset and summarised across the five imputations as median (range). Calibration was assessed with the Hosmer-Lemeshow test across imputed datasets and a calibration plot in the first imputed dataset.

To reduce potential measurement and missing-data bias, clinical information was collected prospectively, and the 90-day mRS was assessed by trained research staff using standardised in-person or telephone follow-up procedures. Missing laboratory data were handled using multiple imputation by chained equations. Potential confounding by measured variables was addressed through multivariable adjustment, and robustness was examined using alternative adjustment sets, re-imputation with 30 datasets and complete-case analysis. Nevertheless, selection bias related to the hospital-based registry population and residual confounding from unmeasured variables cannot be excluded.

### 2.5 Internal validation

To evaluate the robustness of our findings, we implemented three complementary internal validation approaches commonly used for prognostic model assessment:

*Repeated stratified train-validation split*: We performed 100 independent stratified 70/30 splits of the dataset. In each split, the model was trained exclusively on 70% of the data and evaluated on the remaining 30% validation subset within each completed dataset. This process was repeated across all 5 multiply imputed datasets, resulting in 500 separate validation experiments. The primary association between FBG and unfavourable outcome was re-estimated in each validation subset.

*Repeated k-fold cross-validation*: We conducted 20 repetitions of 5-fold cross-validation. In each repetition, the dataset was randomly divided into 5 equal folds, with 4 folds used for model training and 1 fold held out for validation. This process was repeated until all folds had served as the validation set exactly once per repetition, providing repeated cross-validated estimates of model performance within each completed dataset.

*Bootstrap optimism correction*: We generated 500 bootstrap resamples of each imputed dataset (2,500 total resamples). For each resample, we fitted the model and evaluated its performance both in the bootstrap sample (apparent performance) and in the original full dataset (test performance). The difference between these two estimates represents the statistical optimism, which was subtracted from the apparent performance to obtain the final optimism-corrected performance estimate.

All validation analyses used the exact same model specification as the primary analysis. Model performance was primarily assessed using AUC and ΔAUC, and the stability of the FBG effect was evaluated by re-estimating the FBG odds ratio in each validation subset or bootstrap resample.

## 3 Results

### 3.1 Study population and missing data

A total of 1,127 patients aged 18–49 years with acute ischaemic stroke met the inclusion criteria and had 90-day mRS data. Of these, 844 (74.9%) had a favourable outcome (mRS 0–2) and 283 (25.1%) had an unfavourable outcome (mRS ≥ 3).

Missing data were limited to laboratory variables: FBG was missing in 321 patients (28.5%), TG in 255 (22.6%), HDL-C in 236 (20.9%), LDL-C in 222 (19.7%) and TC in 217 (19.3%). Missing data patterns are presented in Supplementary Figure S1. All clinical and treatment variables were complete. Baseline characteristics of the 806 patients with an observed FBG value and the 321 patients without are compared in Supplementary Table S1. The two groups were similar with respect to stroke severity (|SMD| 0.131), level of consciousness (0.082) and ICU admission (0.083), and the primary outcome occurred with similar frequency in patients with and without an observed FBG value (209 of 806, 25.9%, versus 74 of 321, 23.1%; *p* = 0.353). In contrast, patients without a recorded FBG more often had prior diabetes (34.0% versus 11.3%; |SMD| 0.563) and received post-stroke glucose-lowering therapy (43.0% versus 18.9%; |SMD| 0.541), and had higher triglycerides (median 1.74 versus 1.48 mmol/L) and total cholesterol, more frequent pre-stroke medication use (34.0% versus 23.1%) and hypertension, a lower proportion of men and a marginally higher age. Of the 200 patients with a history of diabetes, 109 (54.5%) had no recorded FBG value. In a logistic model of FBG availability (Supplementary Table S2), prior diabetes (OR 2.43, 95% CI 1.55 to 3.82) and post-stroke glucose-lowering therapy (OR 2.05, 95% CI 1.42 to 2.96) were the only variables that remained associated with a missing FBG value after adjustment and correction for multiple testing (false discovery rate *p* = 0.002 for both; older age and female sex showed weaker nominal associations that did not survive this correction), whereas the 90-day outcome was not (OR 0.83, 95% CI 0.58 to 1.17).

### 3.2 Baseline characteristics

The cohort was predominantly male (902/1,127, 80.0%), with a median age of 45.01 years (IQR 40.91–47.91). The median NIHSS score assessed within 24 h of admission was 2 (IQR 1–4). Admission NIHSS scores were higher in patients with an unfavourable 90-day outcome (mRS ≥ 3) than in those with a favourable outcome (mRS 0–2): median 4 (IQR 2–7) versus 2 (IQR 1–3), respectively (Z = -13.038, *p* < 0.001; SMD = 0.761). Patients with an unfavourable outcome also had more impaired consciousness and more frequent ICU admission. Patients with an unfavourable outcome had more severe neurological deficits at baseline, more impaired consciousness and more frequent ICU admission than those with a favourable outcome. Median baseline FBG was higher in patients with an unfavourable outcome (5.72 mmol/L, IQR 4.90–7.22) than in those with a favourable outcome (5.30 mmol/L, IQR 4.70–6.02; *p* < 0.001), whereas TG was lower (1.43 vs. 1.56 mmol/L; *p* = 0.022). In addition, pre-stroke aspirin use was more frequent among patients with unfavourable outcome (7.1% vs. 4.0%; *p* = 0.038). Among acute in-hospital treatments, HUK use was significantly more common in the unfavourable group (67.1% vs. 49.6%; *p* < 0.001), as was thrombolysis (8.5% vs. 4.5%; *p* = 0.011) and glucose-lowering agent use (30.7% vs. 24.1%; *p* = 0.026). Other baseline characteristics were broadly similar between groups (Table 1). The largest between-outcome-group SMDs were observed for continuous admission NIHSS score (0.761), NIHSS category (0.675), GCS category (0.537), FBG (0.462) and post-stroke HUK treatment (0.361).

**Table 1:** The baseline characteristics of the study population by 90-day mRS category.

Variables	Total (*n* = 1,127)	mRS		*Z*/*χ*^2^	*p* value	SMD
		0–2 (*n* = 844)	≥3 (*n* = 283)			
Demographic features						
Male, *n* (%)	902 (80.0)	678 (80.3)	224 (79.2)	0.185	0.667	0.029
Age at onset, years, median (Q1, Q3)	45.01 (40.91, 47.91)	45.00 (40.09, 47.91)	45.07 (40.96, 47.91)	−0.637	0.524	0.038
Clinical characteristics						
*Past medical history*						
Stroke (%)	145 (12.866)	102 (12.085)	43 (15.194)	1.827	0.176	0.091
Hypertension (%)	593 (52.618)	453 (53.673)	140 (49.470)	1.502	0.220	0.084
Diabetes mellitus (%)	200 (17.746)	143 (16.943)	57 (20.141)	1.485	0.223	0.082
CHD (%)	49 (4.348)	33 (3.910)	16 (5.654)	1.550	0.213	0.082
AF (%)	8 (0.710)	5 (0.592)	3 (1.060)	0.161	0.688	0.052
Dyslipidemia (%)	45 (3.993)	32 (3.791)	13 (4.594)	0.356	0.551	0.040
Carotid plaque (%)	5 (0.444)	5 (0.592)	0 (0.000)	0.610	0.435	0.109
Malignancy (%)	11 (0.976)	10 (1.185)	1 (0.353)	0.778	0.378	0.095
*Medication history within 3 months prior to stroke*						
Statins (%)	36 (3.194)	23 (2.725)	13 (4.594)	2.393	0.122	0.100
Aspirin (%)	54 (4.791)	34 (4.028)	20 (7.067)	4.290	0.038	0.133
Clopidogrel (%)	10 (0.887)	8 (0.948)	2 (0.707)	0.000	0.994	0.027
Aspirin + Clopidogrel (%)	13 (1.154)	10 (1.185)	3 (1.060)	0.000	1.000	0.012
ACEIs (%)	48 (4.259)	36 (4.265)	12 (4.240)	0.000	0.986	0.001
ARBs (%)	55 (4.880)	44 (5.213)	11 (3.887)	0.803	0.370	0.064
Diuretics (%)	21 (1.863)	17 (2.014)	4 (1.413)	0.418	0.518	0.046
*β*-blockers (%)	36 (3.194)	29 (3.436)	7 (2.473)	0.635	0.426	0.057
CCBs (%)	125 (11.091)	101 (11.967)	24 (8.481)	2.612	0.106	0.115
Clinical symptom assessment						
Admission NIHSS score, median (Q1, Q3)	2 (1, 4)	2 (1, 3)	4 (2, 7)	−13.038	<0.001	0.761
Stroke severity (%)				−11.036	<0.001	0.675
Mild (NIHSS 0–5)	967 (85.803)	779 (92.299)	188 (66.431)			
Moderate (NIHSS 6–10)	121 (10.736)	59 (6.991)	62 (21.908)			
Severe (NIHSS >10)	39 (3.461)	6 (0.711)	33 (11.661)			
Coma severity (GCS) (%)				−9.108	<0.001	0.537
Mild (GCS 13–15)	1,028(91.216)	807 (95.616)	221 (78.092)			
Moderate (GCS 9–12)	86 (7.631)	37 (4.384)	49 (17.314)			
Severe (GCS ≤ 8)	13 (1.154)	0 (0.000)	13 (4.594)			
Laboratory parameters						
*Lipid metabolism indicators*						
TG (mmol/L)	1.505 (1.048, 2.072)	1.560 (1.060, 2.110)	1.430 (0.995, 1.890)	2.293	0.022	0.201
TC (mmol/L)	4.595 (3.910, 5.380)	4.660 (3.910, 5.380)	4.510 (3.910, 5.350)	0.609	0.542	0.041
LDL-C (mmol/L)	2.850 (2.260, 3.470)	2.870 (2.257, 3.475)	2.810 (2.280, 3.460)	0.081	0.935	0.011
HDL-C (mmol/L)	1.020 (0.880, 1.200)	1.020 (0.880, 1.200)	1.040 (0.900, 1.210)	−1.158	0.247	0.081
*Carbohydrate metabolism indicators*						
FBG (mmol/L)	5.400 (4.740, 6.360)	5.300 (4.700, 6.020)	5.720 (4.900, 7.220)	−4.419	<0.001	0.462
TyG	8.777 (8.368, 9.188)	8.771 (8.368, 9.161)	8.785 (8.404, 9.272)	−0.602	0.547	0.046
*In-hospital management*						
Admission to ICU (yes, %)	48 (4.259)	25 (2.962)	23 (8.127)	13.866	<0.001	0.227
Acute thrombolysis (yes, %)	62 (5.501)	38 (4.502)	24 (8.481)	6.452	0.011	0.162
Statins (yes, %)	1,041 (92.369)	785 (93.009)	256 (90.459)	1.955	0.162	0.093
Aspirin (yes, %)	342 (30.346)	255 (30.213)	87 (30.742)	0.028	0.867	0.011
Clopidogrel (yes, %)	104 (9.228)	72 (8.531)	32 (11.307)	1.951	0.163	0.093
Aspirin + Clopidogrel (yes, %)	624 (55.368)	474 (56.161)	150 (53.004)	0.855	0.355	0.063
HUK (yes, %)	609 (54.037)	419 (49.645)	190 (67.138)	26.112	<0.001	0.361
Post-stroke glucose-lowering therapy (yes, %)	290 (25.732)	203 (24.052)	87 (30.742)	4.963	0.026	0.150

Values are presented as median (Q1, Q3) or *n* (%), as appropriate. mRS, modified Rankin Scale; CHD, coronary heart disease; AF, atrial fibrillation; ACEIs, angiotensin-converting enzyme inhibitors; ARBs, angiotensin II receptor blockers; CCBs, calcium channel blockers; NIHSS, national institutes of health stroke scale; GCS, glasgow coma scale; TG, triglycerides; TC, total cholesterol; HDL-C, high-density lipoprotein cholesterol; LDL-C, low-density lipoprotein cholesterol; FBG, fasting blood glucose; TyG, triglyceride-glucose index; HUK, human urinary kallidinogenase; ICU, intensive care unit. SMD, absolute standardized mean difference; *Z*, standardised Wilcoxon rank-sum statistic; *χ*^2^, chi-squared statistic. SMDs were calculated using observed data; for multilevel categorical variables, the maximum absolute level-specific SMD is shown. Age and admission NIHSS data were available for all 1,127 patients.

### 3.3 Association between FBG and 90-day outcome

Univariable associations are shown in Supplementary Table S3. The adjusted multivariable model is presented in Table 2. After adjustment for NIHSS category, GCS category, prior diabetes history, triglycerides, ICU admission, pre-stroke aspirin use, post-stroke HUK treatment and acute thrombolysis, higher baseline FBG was independently associated with an unfavourable 90-day outcome. Each 1 mmol/L increase in FBG was associated with a 30% increase in the odds of an unfavourable outcome (OR 1.30, 95% CI 1.15 to 1.46; Table 2).

**Table 2:** Multivariable logistic regression models for unfavourable 90-day outcome (mRS ≥ 3).

Variable	Main adjusted model OR (95% CI)	Interaction model OR (95% CI)	Non-diabetic sensitivity OR (95% CI)
FBG (per 1 mmol/L)	1.30 (1.15–1.46)^*^	1.35 (1.20–1.53)^*^	1.35 (1.19–1.53)^*^
Stroke severity: moderate vs. mild	3.54 (2.19–5.71)^*^	3.56 (2.20–5.74)^*^	3.38 (1.98–5.78)^*^
Stroke severity: severe vs. mild	10.43 (3.84–28.35)^*^	10.52 (3.85–28.73)^*^	10.61 (3.51–32.11)^*^
Coma severity: moderate vs. mild	1.82 (1.00–3.33)	1.82 (0.99–3.33)	1.93 (1.01–3.68)^*^
ICU admission	1.90 (0.94–3.83)	1.94 (0.96–3.92)	1.63 (0.76–3.50)
Triglycerides	0.80 (0.65–0.99)^*^	0.78 (0.64–0.96)^*^	0.78 (0.63–0.98)^*^
Prior diabetes history	0.60 (0.35–1.02)	1.83 (0.28–12.21)	–
FBG × prior diabetes	–	0.87 (0.69–1.09)	–
Pre-stroke Aspirin	2.21 (1.18–4.13)^*^	2.21 (1.19–4.12)^*^	1.83 (0.87–3.87)
HUK use	1.96 (1.43–2.68)^*^	1.96 (1.43–2.69)^*^	2.14 (1.50–3.04)^*^
Thrombolysis	1.29 (0.69–2.40)	1.26 (0.67–2.36)	1.41 (0.74–2.68)

The main adjusted model included FBG, NIHSS category, GCS category, prior diabetes history, triglycerides, ICU admission, pre-stroke aspirin use, post-stroke human urinary kallidinogenase treatment and acute thrombolysis. The interaction model used the same covariates and additionally included the FBG × prior diabetes interaction term. In the interaction model, the FBG estimate refers to patients without prior diabetes, and the coefficient for prior diabetes refers to an FBG value of 0 mmol/L and is not interpretable on its own; the stratum-specific FBG estimates are given in Section 3.4. The non-diabetic sensitivity model was restricted to participants without prior diabetes and used the same covariates except prior diabetes history and the interaction term. The FBG odds ratio is reported per 1 mmol/L increase. The reference categories were NIHSS 0–5, GCS 13–15 and absence of the respective binary characteristic or treatment. The severe GCS category showed complete separation because no participant with GCS ≤8 had a favourable outcome; therefore, the corresponding maximum-likelihood odds ratio could not be estimated reliably. The adjustment set was chosen to estimate the association between FBG and outcome; coefficients for other covariates are not causal effect estimates. In particular, the estimate for prior diabetes is conditional on FBG, an intermediate variable, and does not represent the total effect of diabetes on 90-day outcome. OR, odds ratio; CI, confidence interval; FBG, fasting blood glucose; ICU, intensive care unit; HUK, human urinary kallidinogenase. ^*^p < 0.05.

Baseline stroke severity remained the strongest predictor of outcome. Pre-stroke aspirin use (OR 2.21, 95% CI 1.18 to 4.13) and post-stroke HUK treatment (OR 1.96, 95% CI 1.43 to 2.68) were associated with higher odds of an unfavourable outcome, findings most plausibly interpreted as reflecting confounding by indication or baseline severity rather than causal harm. TG showed an inverse association with the primary outcome (OR 0.80, 95% CI 0.65 to 0.99), whereas prior diabetes history was not independently associated with outcome after adjustment for FBG and the other covariates (OR 0.60, 95% CI 0.35 to 1.02). In univariable analysis, prior diabetes was associated with a non-significantly higher risk (OR 1.24, 95% CI 0.88 to 1.74; Supplementary Table S3); the change of direction after adjustment is expected when a variable that lies on the pathway between diabetes and outcome (FBG) is included in the model, and this coefficient should not be read as a protective effect of diabetes. The restricted cubic spline analysis showed no evidence of departure from linearity. Across the five imputed datasets, the likelihood-ratio *p* values for non-linearity had a median of 0.732 (range, 0.666–0.888), and the pooled Wald test for the nonlinear spline terms yielded *p* = 0.992. The estimated dose–response curve increased across the observed FBG range without evidence of a threshold or inflection point (Figure 2), supporting the modelling of FBG as a linear continuous variable.

**Figure 2:**
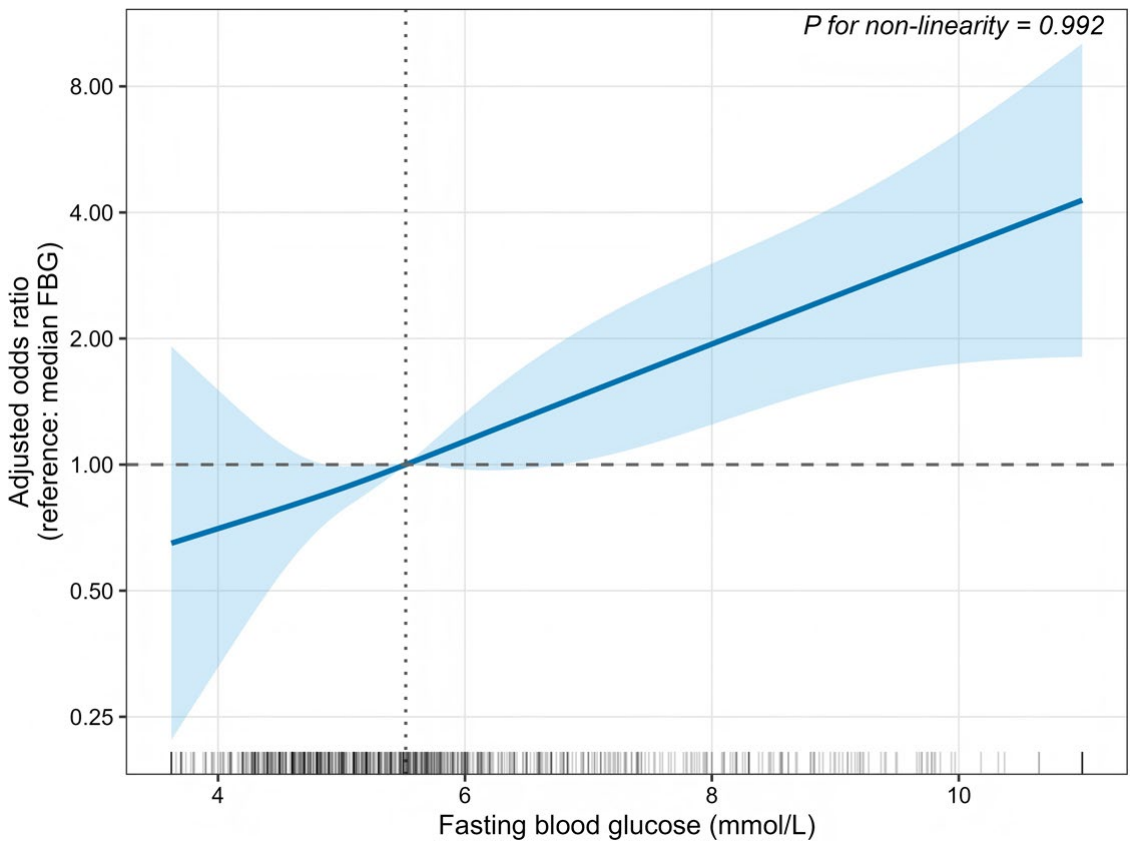
Adjusted dose–response association between baseline fasting blood glucose and unfavourable 90-day outcome. The solid line and shaded area represent the pooled odds ratios and 95% confidence intervals estimated across five imputed datasets using Rubin’s rules. A restricted cubic spline model with four knots at the 5th, 35th, 65th and 95th percentiles of FBG was used. Odds ratios were referenced to the median FBG value of 5.52 mmol/L. The model was adjusted using the primary covariate set. Tick marks indicate the distribution of FBG values. The pooled Wald test showed no evidence of non-linearity (*p* = 0.992).

### 3.4 Effect modification and sensitivity analyses

There was no statistically significant evidence that prior diabetes history modified the association between FBG and outcome (interaction OR 0.87, 95% CI 0.69 to 1.09; *P* for interaction = 0.220). In the sensitivity analysis restricted to patients without prior diabetes, FBG remained independently associated with an unfavourable outcome (OR 1.35, 95% CI 1.19 to 1.53; Table 2). Only 200 patients (17.7%) had a history of diabetes and 57 of them had an unfavourable outcome, so this analysis had limited statistical power; the confidence interval for the interaction term remains compatible with a moderate attenuation of the FBG–outcome association in patients with diabetes, and clinically relevant effect modification cannot be excluded. Stratum-specific estimates derived from the interaction model were OR 1.35 (95% CI 1.20 to 1.53) in patients without prior diabetes and OR 1.18 (95% CI 0.95 to 1.46) in patients with prior diabetes, illustrating that the estimate within the diabetic subgroup is imprecise rather than null. This subgroup estimate should be interpreted with particular caution because 109 of the 200 patients with diabetes had no recorded FBG value (Supplementary Table S1).

The FBG estimate remained consistent across alternative modelling strategies and missing-data approaches (Supplementary Table S4). The OR was 1.30 (95% CI 1.16 to 1.45) in the DAG-minimum model, 1.30 (95% CI 1.15 to 1.47) in the extended model including age and sex, and 1.29 (95% CI 1.14 to 1.46) in the model additionally including post-stroke glucose-lowering therapy. Re-imputation with 30 datasets yielded an OR of 1.32 (95% CI 1.18 to 1.47). Complete-case analysis in 730 patients (188 events and 542 non-events) yielded an OR of 1.38 (95% CI 1.20 to 1.57). The complete-case estimate was slightly larger than the primary estimate (OR 1.38 versus 1.30). This is consistent with the expected behaviour of multiple imputation, which incorporates patients whose FBG was imputed from other variables and therefore tends to shrink estimates towards the null, and with a complete-case sample restricted to patients in whom fasting laboratory testing was actually performed; the two estimates lie well within each other’s confidence intervals and support the same conclusion. The composition of the complete-case sample provides a further explanation: because more than half of the patients with prior diabetes had no recorded FBG value, the complete-case analysis was enriched with patients without diabetes, in whom the FBG effect was larger (OR 1.35 versus 1.18 in patients with diabetes), so a complete-case estimate somewhat above the primary estimate is what would be expected.

### 3.5 Model performance

Across the five imputed datasets, the median AUC of the full adjusted model was 0.748 (range 0.744–0.758), compared with 0.727 (range 0.722–0.729) for the reduced model without FBG (Table 3). The addition of FBG to the reduced model containing all prespecified covariates except FBG resulted in a median increase in AUC of 0.024 (range 0.019–0.031), with a median DeLong *p* value of 0.025 (range 0.006–0.049). The median continuous NRI was 0.306 (range 0.268–0.315), comprising −0.035 (range −0.042 to −0.021) for events and 0.334 (range 0.303–0.351) for non-events; the median IDI was 0.024 (range 0.017–0.029). The gain in the continuous NRI was therefore driven entirely by the non-event component, whereas the event component was negative, indicating that the addition of FBG slightly worsened the assignment of predicted risk among patients who subsequently had an unfavourable outcome. The median Hosmer-Lemeshow *p* value was 0.773 (range 0.454–0.958), consistent with adequate calibration. The calibration plot based on the first imputed dataset showed close agreement between the observed and predicted event rates (Supplementary Figure S2).

**Table 3:** Incremental predictive value of fasting blood glucose across five multiply imputed datasets.

Metric	Median (range) across 5 imputations
AUC, full model (with FBG)	0.748 (0.744–0.758)
AUC, reduced model (without FBG)	0.727 (0.722–0.729)
ΔAUC	0.024 (0.019–0.031)
DeLong P	0.025 (0.006–0.049)
Continuous NRI	0.306 (0.268–0.315)
NRI (events)	−0.035 (−0.042 to −0.021)
NRI (non-events)	0.334 (0.303–0.351)
IDI	0.024 (0.017–0.029)
Calibration (H-L P)	0.773 (0.454–0.958)

Values are summarised as median (range) across the five multiply imputed datasets. The full model included FBG, NIHSS category, GCS category, prior diabetes history, triglycerides, ICU admission, pre-stroke aspirin use, post-stroke HUK treatment and acute thrombolysis. The reduced model contained the same covariates except FBG. AUC, ΔAUC, DeLong p values, continuous net reclassification improvement (cNRI), and integrated discrimination improvement (IDI) were calculated separately within each imputed dataset and were not Rubin-pooled. The continuous NRI is reported together with its event and non-event components, as recommended. The negative event component indicates that adding FBG did not improve the predicted-risk ranking of patients who subsequently had an unfavourable outcome. AUC, area under the receiver operating characteristic curve; NRI, net reclassification improvement; IDI, integrated discrimination improvement; H-L, Hosmer-Lemeshow; FBG, fasting blood glucose.

### 3.6 Internal validation

The repeated 70/30 train-validation split analysis produced a median validation AUC of 0.734 (empirical 2.5th–97.5th percentile interval 0.715–0.762) and a median validation ΔAUC of 0.023 (0.007–0.036). The median validation-set FBG OR was 1.30 (1.10–1.58), and the OR was greater than 1 in 99.6% of validation models.

Repeated 5-fold cross-validation yielded a median AUC of 0.734 (0.724–0.747) and a median ΔAUC of 0.026 (0.017–0.032). Bootstrap validation yielded a median optimism-corrected AUC of 0.739 (0.734–0.748) and an optimism-corrected ΔAUC of 0.024 (0.019–0.030). Across bootstrap resamples, the median FBG OR was 1.30 (1.16–1.45), and all bootstrap estimates were greater than 1. These validation analyses are summarised in Table 4, and the bootstrap distribution of the FBG effect is shown in Supplementary Figure S3.

**Table 4:** Internal validation of the main adjusted model.

Validation procedure	AUC	ΔAUC	FBG effect stability
Repeated 70/30 train-validation split	0.734 (0.715–0.762)	0.023 (0.007–0.036)	Validation-set OR 1.30 (1.10–1.58); OR>1 in 99.6% of validation models
Repeated 5-fold cross-validation	0.734 (0.724–0.747)	0.026 (0.017–0.032)	Directionally consistent incremental value of FBG
Bootstrap optimism correction	0.739 (0.734–0.748)	0.024 (0.019–0.030)	Bootstrap OR 1.30 (1.16–1.45); OR>1 in 100% of bootstrap estimates

Values are medians with empirical 2.5th–97.5th percentile intervals. The repeated train-validation split analysis used 100 stratified random splits. Repeated 5-fold cross-validation used 20 repeats. Bootstrap validation used 500 resamples per imputed dataset. ΔAUC compares the full model with the reduced model without FBG. AUC, area under the receiver operating characteristic curve; FBG, fasting blood glucose; OR, odds ratio.

## 4 Discussion

In this prospective multicentre cohort of young adults with acute ischaemic stroke, higher baseline fasting blood glucose, obtained with the first routine post-admission biochemistry panel, was independently associated with an unfavourable 90-day functional outcome. There was no evidence of non-linearity over the observed FBG range, and the association remained consistent across sensitivity analyses and internal validation procedures. We found no statistically significant evidence that prior diabetes history modified this association, although the diabetic subgroup was small and this test had limited power.

These findings extend previous observations from older or unselected stroke populations to patients with early-onset ischaemic stroke, a group in whom prognostic data on acute glycaemia remain limited. Prior reports in younger cohorts have been limited and heterogeneous (26, 27). In our data, the effect estimates were directionally similar in patients with and without known diabetes, although the smaller diabetic subgroup means that modest interaction effects cannot be excluded.

The absence of detectable non-linearity should not be interpreted as evidence that lowering FBG will improve functional recovery. The present observational analysis estimates the prognostic association between an early FBG measurement and subsequent outcome, whereas randomised trials evaluate the effect of glucose-lowering strategies initiated after stroke onset. Higher FBG may reflect acute metabolic stress, stroke severity, impaired tissue metabolism and underlying vulnerability, in addition to any direct adverse effects of hyperglycaemia (20, 48, 49). Treatment initiated after ischaemic injury has occurred may not reverse the pathways leading to disability, and any potential benefit may be offset by hypoglycaemia, glycaemic variability or treatment-related risk. Randomised trials, including SHINE, GIST-UK and TEXAIS, have not demonstrated a clear improvement in functional outcome with intensive or protocolised glucose lowering (29, 30, 50). These findings do not contradict the prognostic association observed in our cohort. Our results therefore support FBG as a readily available prognostic correlate but do not establish a glucose-lowering target or imply that intensive glucose lowering improves functional outcome.

Several biological mechanisms may explain the association between higher glucose and worse outcome after ischaemic stroke. Acute hyperglycaemia may exacerbate anaerobic glycolysis and lactic acidosis in ischaemic tissue, increase oxidative stress, impair endothelial function and disrupt the blood–brain barrier (20, 48, 49). Because the analysed exposure was an early in-hospital fasting glucose measure recorded at baseline rather than a purely random arrival glucose value, it may reflect a more stable early metabolic disturbance rather than transient prehospital stress alone.

Some secondary associations require cautious interpretation. The inverse association between TG and poor outcome is consistent with reports of a possible lipid paradox in stroke, but the finding may reflect nutritional status, reverse causation or residual confounding rather than a protective effect (42, 51). Likewise, the associations of pre-stroke aspirin use and HUK treatment with poorer outcome are most likely explained by confounding by indication and should not be interpreted causally (43–45).

The adjusted coefficient for prior diabetes deserves separate comment. In univariable analysis, prior diabetes was weakly and non-significantly associated with a higher risk of an unfavourable outcome (OR 1.24, 95% CI 0.88 to 1.74), whereas in the FBG-adjusted model the point estimate was inverted (OR 0.60, 95% CI 0.35 to 1.02). Chronic diabetes acts on post-stroke outcome largely through glycaemic status, so that early fasting glucose lies on the pathway between diabetes and functional recovery. Conditioning on FBG removes this pathway and leaves a comparison between patients with and without diabetes who present with the same fasting glucose value. Such patients are not exchangeable: a patient with known and treated diabetes whose admission fasting glucose is normal differs systematically from a patient without diabetes at the same value with respect to glucose-lowering treatment, prior vascular care and the metabolic meaning of that value. The inverted estimate is therefore best understood as an instance of the Table 2 fallacy, in which coefficients of covariates included to adjust the exposure of interest are given a causal reading that the model does not support (46). We make no causal claim about diabetes on the basis of this model (see the footnote to Table 2), and a model intended to estimate the effect of prior diabetes would require a different adjustment set that does not condition on FBG. The instability of this coefficient across missing-data approaches supports this reading: the adjusted odds ratio for prior diabetes was 0.60 (95% CI 0.35 to 1.02) with five imputed datasets, 0.57 (95% CI 0.34 to 0.97) after re-imputation with 30 datasets, and 0.97 (95% CI 0.51 to 1.84) in the complete-case analysis, whereas the FBG estimate was stable across the same analyses. The pattern of missingness reinforces this reading. Of the 200 patients with prior diabetes, 109 (54.5%) had no recorded fasting glucose, so the adjusted diabetes coefficient rests substantially on imputed exposure values in precisely the subgroup in which it is estimated.

The incremental predictive value of FBG was modest and not uniform across metrics. Adding FBG to a model that already contained stroke severity, level of consciousness and the other prespecified covariates increased the AUC by 0.024 (median DeLong *P* 0.025, range across imputations 0.006–0.049) and produced an IDI of 0.024. The continuous NRI of 0.306 should not be read as evidence of substantial clinical gain: its event component was negative (−0.035), so the ranking of predicted risk among patients who subsequently had an unfavourable outcome was marginally worse after FBG was added, and the overall value was generated entirely by the non-event component (0.334). Category-free reclassification indices are known to overstate the incremental value of a new marker and should be reported separately for events and non-events, as we have done (52). Taken together, these analyses support a reproducible and independent association between baseline FBG and 90-day outcome, but they do not establish that FBG materially improves individual risk prediction beyond routinely available clinical variables.

This study has several limitations. First, the observational design cannot establish causality. The association may be partly mediated by infarct severity or the acute stress response rather than reflecting a direct detrimental effect of hyperglycaemia, and residual confounding cannot be excluded, particularly because smoking, alcohol use and body mass index could not be adequately incorporated. Second, the exposure could be characterised only partly. Glycated haemoglobin was unavailable, so acute stress hyperglycaemia could not be distinguished from chronic hyperglycaemia. The registry did not record the time of blood sampling, so the interval from symptom onset to measurement could not be quantified or adjusted for, and in-hospital glucose-lowering therapy may have preceded the fasting sample in some patients. In patients sampled after stress hyperglycaemia had partly resolved, or after treatment had begun, the measured FBG would understate admission glycaemia; such measurement error would generally be expected to attenuate rather than generate the observed association, although misclassification related to admission delay cannot be excluded. Third, FBG was missing in 28.5% of patients, and its availability was not random (Supplementary Tables S1, S2). The selection was metabolic rather than prognostic: availability was unrelated to stroke severity, level of consciousness, ICU admission and the 90-day outcome, whereas patients with prior diabetes and those who received glucose-lowering therapy were about twice as likely to have no recorded fasting value, plausibly because their glucose was monitored by point-of-care or non-fasting measurements. Because these predictors were included in the imputation model and missingness was unrelated to the outcome, the missing-at-random assumption is reasonable, and the primary estimate was stable across imputation and complete-case analyses; the assumption nevertheless cannot be verified empirically. Fourth, heterogeneity of the association could be examined only partially. The test for interaction with prior diabetes was based on 200 patients with diabetes (57 events), of whom 109 had no recorded FBG value, so it was underpowered and depended heavily on imputed exposure values; the absence of a significant interaction should not be taken as evidence of a uniform effect, and the estimates within the diabetic stratum should be regarded as exploratory. In addition, TOAST subtype data were too incomplete to assess effect modification by stroke aetiology. Finally, validation was conducted within the CASTOR registry rather than in a separately recruited external cohort, and selection bias related to the hospital-based population cannot be excluded. The findings should therefore be generalised with caution beyond hospitalised Chinese adults aged 18–49 years with acute ischaemic stroke admitted within 7 days of symptom onset and treated in comparable routine stroke-care settings.

## 5 Conclusion

Higher baseline fasting blood glucose was independently associated with an unfavourable 90-day functional outcome in young adults with acute ischaemic stroke. The association showed no evidence of non-linearity and was consistent across sensitivity analyses and internal validation procedures. No statistically significant interaction with prior diabetes history was detected, although power to detect effect modification was limited. The incremental predictive value of FBG beyond the prespecified covariate model was small and inconsistent across reclassification metrics, the event component of the net reclassification improvement being negative. Baseline FBG is therefore best regarded as a readily available correlate of 90-day prognosis rather than a validated tool for individual risk stratification; external validation with standardised sampling times is required.

## Data Availability

The raw data supporting the conclusions of this article will be made available by the authors, without undue reservation.
