# Supplementary material for: Early in-hospital fasting blood glucose and 90-day functional outcome in early-onset ischaemic stroke: a multicentre prospective cohort study
Source: Front Neurol. 2026 Oct 30;17:1895654. doi: 10.3389/fneur.2026.1895654 (PMC13759401; doi:10.3389/fneur.2026.1895654)
Supplement: Supplementary file 1 [file Supplementary_file_1.DOCX]

**Early in-hospital fasting blood glucose and 90-day functional outcome in early-onset ischaemic stroke: a multicentre prospective cohort study**

**Supplementary materials**

All supplementary materials are presented in the order in which they are cited in the main text.

**Figure S1 Missing data patterns across key analysis variables**

Aggregation plot showing the proportion of missing data (left panel) and the patterns of missingness (right panel) for all key analysis variables. Variables are sorted by missing rate in descending order. Blue segments indicate observed values; red segments indicate missing values. The right panel displays the frequency of each unique missing data pattern. Abbreviations: FBG, fasting blood glucose; TG, triglycerides; TC, total cholesterol; HDL-C, high-density lipoprotein cholesterol; LDL-C, low-density lipoprotein cholesterol.

**Table S1 Comparison of baseline characteristics between patients with an observed fasting blood glucose value and patients with a missing fasting blood glucose value**

| **Variable** | **FBG observed (n=806)** | **FBG missing (n=321)** | **\|SMD\|** | **P value** |
| --- | --- | --- | --- | --- |
| **Demographic features** |  |  |  |  |
| Age at stroke onset, years, median (Q1, Q3) | 45.00 (39.94, 47.10) | 45.08 (41.98, 47.94) | 0.194 | 0.023 |
| Male, n (%) | 660 (81.9) | 242 (75.4) | 0.159 | 0.017 |
| **Past medical history** |  |  |  |  |
| Prior stroke, n (%) | 93 (11.5) | 52 (16.2) | 0.135 | 0.044 |
| Hypertension, n (%) | 405 (50.2) | 188 (58.6) | 0.168 | 0.014 |
| Diabetes mellitus, n (%) | 91 (11.3) | 109 (34.0) | 0.563 | <0.001 |
| Coronary heart disease, n (%) | 33 (4.1) | 16 (5.0) | 0.043 | 0.617 |
| Atrial fibrillation, n (%) | 8 (1.0) | 0 (0.0) | 0.142 | 0.114 |
| Dyslipidaemia, n (%) | 35 (4.3) | 10 (3.1) | 0.065 | 0.435 |
| Carotid plaque, n (%) | 2 (0.2) | 3 (0.9) | 0.090 | 0.143 |
| Malignancy, n (%) | 7 (0.9) | 4 (1.2) | 0.037 | 0.519 |
| **Medication history within 3 months prior to stroke** |  |  |  |  |
| Any pre-stroke medication, n (%) | 186 (23.1) | 109 (34.0) | 0.243 | <0.001 |
| Statins, n (%) | 21 (2.6) | 15 (4.7) | 0.111 | 0.111 |
| Aspirin, n (%) | 34 (4.2) | 20 (6.2) | 0.091 | 0.203 |
| Clopidogrel, n (%) | 5 (0.6) | 5 (1.6) | 0.090 | 0.159 |
| Aspirin + clopidogrel, n (%) | 8 (1.0) | 5 (1.6) | 0.050 | 0.536 |
| ACEIs, n (%) | 29 (3.6) | 19 (5.9) | 0.109 | 0.115 |
| ARBs, n (%) | 34 (4.2) | 21 (6.5) | 0.103 | 0.139 |
| Diuretics, n (%) | 11 (1.4) | 10 (3.1) | 0.118 | 0.086 |
| β-blockers, n (%) | 19 (2.4) | 17 (5.3) | 0.154 | 0.019 |
| CCBs, n (%) | 86 (10.7) | 39 (12.1) | 0.047 | 0.543 |
| **Clinical symptom assessment** |  |  |  |  |
| Stroke severity, mild (NIHSS 0–5), n (%) | 683 (84.7) | 284 (88.5) | 0.131 | 0.126 |
| Stroke severity, moderate (NIHSS 6–10), n (%) | 90 (11.2) | 31 (9.7) | 0.131 | 0.126 |
| Stroke severity, severe (NIHSS >10), n (%) | 33 (4.1) | 6 (1.9) | 0.131 | 0.126 |
| Coma severity, GCS 13–15, n (%) | 730 (90.6) | 298 (92.8) | 0.082 | 0.487 |
| Coma severity, GCS 9–12, n (%) | 66 (8.2) | 20 (6.2) | 0.082 | 0.487 |
| Coma severity, GCS ≤8, n (%) | 10 (1.2) | 3 (0.9) | 0.082 | 0.487 |
| ICU admission, n (%) | 38 (4.7) | 10 (3.1) | 0.083 | 0.300 |
| **Laboratory parameters** |  |  |  |  |
| Triglycerides, mmol/L, median (Q1, Q3) [n=730/142] | 1.48 (1.02, 1.99) | 1.74 (1.17, 2.44) | 0.350 | <0.001 |
| Total cholesterol, mmol/L, median (Q1, Q3) [n=750/160] | 4.58 (3.89, 5.35) | 4.73 (4.10, 5.63) | 0.215 | 0.024 |
| HDL-C, mmol/L, median (Q1, Q3) [n=737/154] | 1.02 (0.88, 1.21) | 1.02 (0.88, 1.19) | 0.043 | 0.615 |
| LDL-C, mmol/L, median (Q1, Q3) [n=745/160] | 2.84 (2.26, 3.49) | 2.90 (2.29, 3.42) | 0.049 | 0.610 |
| **Acute and in-hospital treatment** |  |  |  |  |
| Acute thrombolysis, n (%) | 48 (6.0) | 14 (4.4) | 0.072 | 0.360 |
| Aspirin, n (%) | 240 (29.8) | 102 (31.8) | 0.043 | 0.557 |
| Clopidogrel, n (%) | 75 (9.3) | 29 (9.0) | 0.009 | 0.978 |
| Aspirin + clopidogrel, n (%) | 450 (55.8) | 174 (54.2) | 0.033 | 0.668 |
| Statins, n (%) | 747 (92.7) | 294 (91.6) | 0.041 | 0.618 |
| HUK, n (%) | 425 (52.7) | 184 (57.3) | 0.092 | 0.184 |
| Glucose-lowering therapy, n (%) | 152 (18.9) | 138 (43.0) | 0.541 | <0.001 |
| **Outcome** |  |  |  |  |
| Unfavourable 90-day outcome (mRS ≥3), n (%) | 209 (25.9) | 74 (23.1) | 0.067 | 0.353 |

**Values are n (%) or median (Q1, Q3). The comparison includes all 1,127 patients in the analysis cohort; FBG was observed in 806 patients and missing in 321 (28.5%). Absolute standardized mean differences (|SMD|) were calculated from observed data; for multilevel categorical variables the maximum absolute level-specific SMD is shown, and |SMD| ≥0.10 was considered potentially meaningful. P values are from the Wilcoxon rank-sum test for continuous variables and the χ² or Fisher exact test for categorical variables. Values in square brackets after a laboratory variable give the number of patients with that measurement available in each group, because the laboratory variables were frequently missing together. Patients without a recorded FBG did not differ from those with a recorded FBG in stroke severity, level of consciousness, ICU admission or the 90-day outcome, but were substantially more likely to have prior diabetes and to receive post-stroke glucose-lowering therapy. Abbreviations: FBG, fasting blood glucose; NIHSS, National Institutes of Health Stroke Scale; GCS, Glasgow Coma Scale; ICU, intensive care unit; HUK, human urinary kallidinogenase; HDL-C, high-density lipoprotein cholesterol; LDL-C, low-density lipoprotein cholesterol; mRS, modified Rankin Scale; ACEIs, angiotensin-converting enzyme inhibitors; ARBs, angiotensin II receptor blockers; CCBs, calcium channel blockers; SMD, standardized mean difference.**

**Table S2 Logistic model of the probability that the fasting blood glucose value was missing**

| **Variable** | **OR (95% CI) for a missing FBG value** | **P value** | **FDR P** |
| --- | --- | --- | --- |
| Prior diabetes | 2.429 (1.546-3.816) | <0.001 | 0.002 |
| Post-stroke glucose-lowering therapy | 2.050 (1.418-2.965) | <0.001 | 0.002 |
| Age at stroke onset, per year | 1.034 (1.006-1.062) | 0.016 | 0.178 |
| Male sex | 0.707 (0.503-0.993) | 0.045 | 0.365 |
| NIHSS >10 vs 0–5 | 0.356 (0.120-1.056) | 0.063 | 0.365 |
| Post-stroke HUK | 1.302 (0.975-1.738) | 0.074 | 0.365 |
| Dyslipidaemia | 0.487 (0.219-1.082) | 0.077 | 0.365 |
| Pre-stroke β-blocker | 1.869 (0.861-4.057) | 0.114 | 0.470 |
| Pre-stroke CCB | 0.635 (0.348-1.157) | 0.138 | 0.506 |
| Pre-stroke clopidogrel | 2.618 (0.621-11.039) | 0.190 | 0.627 |
| NIHSS 6–10 vs 0–5 | 0.709 (0.410-1.225) | 0.218 | 0.653 |
| Any pre-stroke medication | 1.415 (0.782-2.559) | 0.251 | 0.691 |
| Unfavourable 90-day outcome (mRS ≥3) | 0.826 (0.582-1.172) | 0.284 | 0.720 |
| GCS 9–12 vs 13–15 | 1.343 (0.692-2.607) | 0.384 | 0.839 |
| Pre-stroke statin | 1.494 (0.565-3.951) | 0.418 | 0.839 |
| Coronary heart disease | 0.751 (0.369-1.530) | 0.431 | 0.839 |
| Post-stroke statin | 0.802 (0.463-1.390) | 0.432 | 0.839 |
| ICU admission | 0.755 (0.348-1.641) | 0.479 | 0.878 |
| Pre-stroke aspirin + clopidogrel | 0.647 (0.164-2.563) | 0.536 | 0.896 |
| Pre-stroke diuretic | 1.374 (0.494-3.818) | 0.543 | 0.896 |
| Post-stroke aspirin | 1.134 (0.564-2.282) | 0.724 | 0.905 |
| Prior stroke | 1.080 (0.691-1.689) | 0.736 | 0.905 |
| Pre-stroke aspirin | 0.863 (0.363-2.049) | 0.739 | 0.905 |
| Post-stroke aspirin + clopidogrel | 0.893 (0.450-1.775) | 0.747 | 0.905 |
| Pre-stroke ARB | 0.896 (0.440-1.825) | 0.762 | 0.905 |
| Malignancy | 1.215 (0.324-4.549) | 0.773 | 0.905 |
| GCS ≤8 vs 13–15 | 1.266 (0.254-6.308) | 0.774 | 0.905 |
| Post-stroke clopidogrel | 0.966 (0.434-2.150) | 0.932 | 0.997 |
| Acute thrombolysis | 1.011 (0.481-2.123) | 0.978 | 0.997 |
| Pre-stroke ACEI | 0.990 (0.473-2.070) | 0.978 | 0.997 |
| Hypertension | 0.999 (0.613-1.629) | 0.997 | 0.997 |

**Odds ratios are for having a missing rather than an observed FBG value, from a single logistic model containing all completely observed baseline variables and the 90-day outcome (n=1,127; 321 missing FBG values); atrial fibrillation and carotid plaque were not included because fewer than 10 patients had these conditions. Terms are ordered by P value. Acute alteplase was omitted because it is nested within the acute thrombolysis indicator. FDR P values were obtained with the Benjamini-Hochberg procedure. Prior diabetes and post-stroke glucose-lowering therapy were the only variables that remained associated with missingness after adjustment and correction for multiple testing; age and male sex showed nominal associations (P<0.05) that did not survive this correction; the 90-day outcome was not associated with missingness, which supports the missing-at-random assumption made in the primary analysis. Abbreviations: OR, odds ratio; CI, confidence interval; FBG, fasting blood glucose; NIHSS, National Institutes of Health Stroke Scale; GCS, Glasgow Coma Scale; ICU, intensive care unit; ACEI, angiotensin-converting enzyme inhibitor; ARB, angiotensin II receptor blocker; CCB, calcium channel blocker; HUK, human urinary kallidinogenase; mRS, modified Rankin Scale; FDR, false discovery rate.**

**Table S3 Univariable associations between baseline characteristics and unfavourable 90-day outcome (mRS≥3), pooled across five multiply imputed datasets**

| **Variables** | **OR (95% CI)** | **P value** |
| --- | --- | --- |
| Stroke severity (moderate) | 4.35 (2.95-6.44) | <0.001 |
| Stroke severity (severe) | 22.79 (9.40-55.23) | <0.001 |
| Coma severity (moderate) | 4.84 (3.08-7.60) | <0.001 |
| Post-stroke HUK (yes) | 2.07 (1.56-2.75) | <0.001 |
| Admission to ICU (yes) | 2.90 (1.62-5.20) | <0.001 |
| FBG (mmol/L) | 1.21 (1.09-1.35) | 0.001 |
| Acute thrombolysis (yes) | 1.97 (1.16-3.34) | 0.013 |
| TG (mmol/L) | 0.80 (0.66-0.96) | 0.016 |
| Post-stroke glucose-lowering therapy (yes) | 1.40 (1.04-1.89) | 0.026 |
| Aspirin use history (yes) | 1.81 (1.02-3.20) | 0.041 |
| CCBs use history (yes) | 0.68 (0.43-1.09) | 0.108 |
| Statins use history (yes) | 1.72 (0.86-3.44) | 0.126 |
| Post-stroke Statins (yes) | 0.71 (0.44-1.15) | 0.164 |
| Post-stroke Clopidogrel (yes) | 1.37 (0.88-2.12) | 0.164 |
| Prior stroke (yes) | 1.30 (0.89-1.92) | 0.178 |
| Coronary heart disease (yes) | 1.47 (0.80-2.72) | 0.216 |
| Hypertension (yes) | 0.85 (0.65-1.11) | 0.221 |
| Diabetes mellitus (yes) | 1.24 (0.88-1.74) | 0.224 |
| Malignancy (yes) | 0.30 (0.04-2.32) | 0.247 |
| Post-stroke Aspirin + Clopidogrel (yes) | 0.88 (0.67-1.15) | 0.355 |
| ARBs use history (yes) | 0.74 (0.37-1.45) | 0.372 |
| Atrial fibrillation (yes) | 1.80 (0.43-7.58) | 0.424 |
| β-blockers use history (yes) | 0.71 (0.31-1.65) | 0.428 |
| HDL-C (mmol/L) | 1.25 (0.70-2.24) | 0.448 |
| Diuretics use history (yes) | 0.70 (0.23-2.09) | 0.520 |
| Any pre-stroke medication (yes) | 0.90 (0.66-1.23) | 0.524 |
| Dyslipidaemia (yes) | 1.22 (0.63-2.36) | 0.552 |
| Age at stroke onset, per year | 1.01 (0.98-1.03) | 0.574 |
| TC (mmol/L) | 0.97 (0.84-1.11) | 0.630 |
| Sex (Male) | 0.93 (0.67-1.30) | 0.668 |
| Clopidogrel use history (yes) | 0.74 (0.16-3.53) | 0.709 |
| Aspirin+Clopidogrel use history (yes) | 0.89 (0.24-3.27) | 0.865 |
| Post-stroke Aspirin (yes) | 1.03 (0.77-1.37) | 0.867 |
| Triglyceride–glucose index | 0.98 (0.76-1.28) | 0.898 |
| LDL-C (mmol/L) | 1.01 (0.83-1.24) | 0.901 |
| Coma severity (severe) | Not estimable owing to complete separation | 0.967 |
| Carotid plaque (yes) | Not estimable owing to complete separation | 0.973 |
| ACEIs use history (yes) | 0.99 (0.51-1.94) | 0.986 |

Odds ratios for continuous variables are reported per one-unit increase unless otherwise specified. Reference categories for categorical variables are indicated in the variable labels. Estimates that could not be obtained because of complete separation are reported as not estimable. Abbreviations: OR, odds ratio; CI, confidence interval; HUK, human urinary kallidinogenase; ICU, intensive care unit; FBG, fasting blood glucose; TG, triglycerides; CCBs, calcium channel blockers; CHD, coronary heart disease; DM, diabetes mellitus; ARBs, angiotensin II receptor blockers; AF, atrial fibrillation; HDL-C, high-density lipoprotein cholesterol; TC, total cholesterol; LDL-C, low-density lipoprotein cholesterol; TyG, triglyceride-glucose index; ACEIs, angiotensin-converting enzyme inhibitors.

**Table S4 Sensitivity analyses of the association between fasting blood glucose and unfavourable 90-day outcome**

| **Sensitivity analysis** | **FBG OR (95% CI)** | **P value** |
| --- | --- | --- |
| Primary model, m=5 imputation | 1.298 (1.152–1.462) | <0.001 |
| DAG-minimum model | 1.299 (1.162–1.452) | <0.001 |
| Extended model (+age, +sex) | 1.301 (1.153–1.467) | <0.001 |
| Model including post-stroke glucose-lowering therapy | 1.291 (1.140–1.462) | <0.001 |
| Re-imputation, m=30 | 1.316 (1.180–1.467) | <0.001 |
| Complete-case analysis (n=730) | 1.377 (1.204–1.575) | <0.001 |

Values are odds ratios per 1 mmol/L increase in fasting blood glucose. The primary model was adjusted for NIHSS category, GCS category, prior diabetes history, triglycerides, ICU admission, pre-stroke aspirin use, post-stroke HUK treatment and acute thrombolysis. The DAG-minimum model included FBG, NIHSS category, GCS category, prior diabetes history and triglycerides. The extended model additionally included age and sex. The treatment sensitivity model additionally included post-stroke glucose-lowering therapy. Complete-case analysis included 730 patients (188 events and 542 non-events). Abbreviations: FBG, fasting blood glucose; DAG, directed acyclic graph; NIHSS, National Institutes of Health Stroke Scale; GCS, Glasgow Coma Scale; ICU, intensive care unit; HUK, human urinary kallidinogenase.

**Figure S2 Calibration plot for the primary adjusted logistic regression model**

Calibration plot based on the first imputed dataset. The observed event rate is plotted against the mean predicted probability across deciles of predicted risk. The dashed diagonal line indicates perfect calibration, and point size is proportional to the number of patients in each decile. The plot shows close agreement between observed and predicted event rates, consistent with adequate calibration of the primary adjusted model. Abbreviations: FBG, fasting blood glucose; H-L, Hosmer-Lemeshow.

**Figure S3 Bootstrap distribution of the FBG odds ratio**

The density curve shows bootstrap estimates of the FBG odds ratio from the main adjusted model across multiply imputed datasets. The vertical dashed line indicates OR=1. The distribution was centred above 1, supporting the stability and directionality of the association between higher FBG and unfavourable 90-day outcome. FBG, fasting blood glucose; OR, odds ratio.
